# Patient-reported experience measures for people living with dementia: A scoping review

**DOI:** 10.1177/14713012241272823

**Published:** 2024-08-08

**Authors:** Madison Chapman, Rachel Milte, Suzanne Dawson, Kate Laver

**Affiliations:** Caring Futures Institute, College of Nursing and Health Sciences, 1065 Flinders University, Australia; Caring Futures Institute, College of Nursing and Health Sciences, 1065 Flinders University, Australia; Rehabilitation, Aged and Palliative Care Service, 568639 Southern Adelaide Local Health Network, Australia

**Keywords:** patient-reported experience measures, dementia, quality of care

## Abstract

The prevalence of dementia is increasing globally, with an estimated 139 million people expected to be living with dementia by 2050. Across numerous countries, substandard care for people with dementia is evident, with quality improvement needed. Recently, a focus on patient-reported experience measures (PREMs) has been utilised in healthcare services as a method of evaluating the care experiences provided and determining areas of improvement. The literature is scarce regarding the feasibility and acceptability of implementing PREMs with people with moderate to advanced dementia. This scoping review aimed to identify PREMs that have been used with vulnerable populations including people with cognitive impairment, mental health concerns, and children, outline dimensions included, and determine adaptions made to the PREMs to improve acceptability of the instruments for vulnerable populations. A database search of Medline was conducted to identify 36 studies including 32 PREMs. The PREMs identified covered a range of dimensions, most frequently care effectiveness, care environment, and patient involvement. The most common adaption to the PREMs was simplification of wording and sentence structure. Several measures conflated patient outcomes and patient satisfaction with patient experience, limiting utility for improving patient experience specifically. While several PREMs have been used with people with dementia, challenges in their implementation and their applicability to specific settings limit their use more broadly. Evidently, there is a need for development of a PREM for people with moderate to advanced dementia that is applicable across healthcare settings and is appropriately adapted for varying cognitive and communicative barriers.

## Introduction

By current global estimates, the number of people living with dementia is expected to grow from 55 million in 2019 to 139 million people by 2050 (Long et al., 2023). Consequently, dementia has been recognised as a health priority in Australia (Australian Health Ministers' Advisory Council, 2015) and globally (World Health Organization, 2012). With a growing number of people living with dementia, there is greater need for dementia health and aged care services. Globally, the quality of care provided for people living with dementia has raised concern, with the 2022 World Alzheimer Report noting that across multiple countries, particularly lower- and middle-income countries, the burden of dementia care lay predominantly with family members and that there was a need for governments to develop national dementia plans and improve funding for dementia care (Gauthier, 2022). Within Australia, the Royal Commission into Aged Care Quality and Safety (2021) uncovered “persistent” substandard care for people living with dementia, highlighting dementia care as one of four concerns for immediate attention.

Over the past two decades, there has been increasing attention upon understanding quality of care through measures of patient experience (Ahmed et al., 2014). This understanding of the patient experience is a necessary step in providing person-centred care (often described as care that promotes partnership and collaboration between healthcare professionals and patients, acknowledges patients’ preferences and values, and operates flexibly and in consideration of individualistic needs) (Delaney, 2018). Person-centred care, across healthcare domains, is associated with improved patient outcomes (Olsson et al., 2014), increased patient wellbeing and quality of life (Apers et al., 2018; Gameiro et al., 2013; Kuipers et al., 2019), improved adherence to treatment plans (Roumie et al., 2011; Thompson & McCabe, 2012), and cost-effectiveness of services (Pirhonen et al., 2020). In multiple countries, assessment of patient experience, operationalised through administration of patient-reported experience measures (PREMs), is being recognised as an important metric in measuring and monitoring quality of healthcare and as a key component in quality improvement (Gilmore et al., 2023; Holt, 2019).

Patient experience, while related, is conceptually different to patient satisfaction. While patient satisfaction in healthcare settings began gaining attention as early as the 1950s (Melchert, 2011), patient satisfaction lacks utility in quality improvement due to frequent ceiling effects and an emphasis upon patient’s expectations, thus being more subjective and open to bias (Bull, 2021). Instead, PREMs provide a more deliberate method of delineating aspects of the healthcare experience to encourage tangible improvements in healthcare by asking objective questions of the patient (Bull, 2021).

Current models of dementia care emphasise the importance of relationship-centred care, where the involvement of healthcare professionals, family members, and the person with dementia are considered (Nolan, 2006; Nolan et al., 2004). Relationship-centred care ensures that family members are included in discussions of care however, it also risks the reliance on family members’ experiences being substituted in as proxy measures for quality of life and care experience due to cognitive impairment and communication barriers making the perspectives of people living with dementia difficult to obtain (Griffiths et al., 2020). While the experiences of family members and carers are valuable, the literature has demonstrated clear differences in patient and proxy reports of quality of life for people living with dementia (Griffiths et al., 2020; Hutchinson et al., 2022). Similarly, within the context of proxy reported PREMs, parents often report more positive healthcare experiences than children in paediatric settings, and there is often misalignment between parents and children regarding the factors considered most important in the healthcare context (Bal et al., 2020; Boss & Thompson, 2012; McGrath et al., 2024). As outlined in the global action plan on the public health response to dementia (World Health Organization, 2017), there is a need to empower and engage with people living with moderate to advanced dementia to ensure that their voices, and not just proxies, are included in considerations of how to improve dementia care. A number of quality of life tools have now been developed and/or validated for people with dementia (Keetharuth et al., 2022; Lay et al., 2024). While these tools are important in understanding wellbeing more broadly, a focus on the development and validation of PREMs for people with moderate to advanced dementia will provide more specific data on their healthcare experiences.

As part of our program of research we aim to develop, pilot, and validate a PREM for people living with moderate to advanced dementia as we found that so far no psychometrically-sound PREM exists for this population across healthcare services. As an initial step in the development process, we conducted a scoping review to identify PREMs that have been used with vulnerable populations, such as in paediatric, mental health, and disability settings as these populations likely require PREMs to be adapted for cognitive ability, communication issues, and/or experiences of mental fatigue. We aimed to examine the following research questions:

  1. What are the characteristics of PREMs that have been used with vulnerable populations?
  2. What dimensions of patient experience are covered by these PREMs?
  3. What adaptions have been made to the PREMs to account for cognitive and communicative barriers in their application with vulnerable populations?

## Method

### Eligibility criteria

The scoping review included studies written in English that involved the use of PREMs with populations who may have impaired or developing cognitive or communicative ability. We included populations comprising: children less than 12 years, people with mental health concerns, or people with cognitive impairment. Studies that included these populations but did not report results for these cohorts specifically were excluded (e.g., combined children and parent measures in reporting results). Instruments were considered PREMs if they included objective questions (e.g., “Did you have to wait longer than half an hour for your appointment?”) rather than purely subjective questions (e.g., “How satisfied were you with the wait time for your appointment?”). Studies that included the use of PREMs with vulnerable populations, even if the main outcome was not developing or validating PREMs with a certain cohort, were included to ensure the breadth of the review. All study methodologies were considered acceptable, as were both published and unpublished studies (e.g., theses).

### Information sources

This scoping review was conducted according to Arksey and O’Malley’s (2005) methodological framework for scoping reviews and is reported in accordance with the Preferred Reporting Items for Systematic Reviews and Meta-Analyses Extension for Scoping Reviews (PRISMA-ScR) Checklist (Tricco et al., 2018). The study protocol is available upon request from the corresponding author. The selected database for the search was Medline and following the researchers agreeing upon the search strategy (available in Table S1, as supplemental material), the search was conducted on studies published between 1946 and September 2023. The researchers also sourced studies from manual searching of the grey literature on Google Scholar and scanning the reference lists of relevant studies.

### Selection of sources of evidence

The titles and abstracts of all studies were screened by two authors. Disagreements were discussed to reach consensus. Full-text articles were then reviewed by both authors to decide if studies met inclusion criteria. Disagreements were again discussed to reach the final selection of studies to be included in the scoping review.

### Data charting process

Data extraction was performed by one author, using an Excel spreadsheet, which was reviewed by a second author. The data charting process aimed to capture the characteristics of the included studies that would assist in answering the research questions. Extracted data included study details (authors, year of publication, country/countries the study was conducted in, language(s) of the PREM used, study design, study outcome), population information (category, target population, number of participants, adaptions to the PREM for the population), characteristics of the PREM and its use (number of items, dimensions, response options, mode of administration, recall period), and psychometric properties (internal consistency, test-retest reliability, face validity, content validity, construct validity, criterion validity, and responsiveness).

### Critical appraisal of individual sources of evidence

According to the PRISMA-ScR checklist (Tricco et al., 2018), critical appraisal is an optional step as the purpose of the scoping review is to provide an overview of available evidence. Due to the paucity of PREMs validated with vulnerable populations, we chose not to conduct a critical appraisal to ensure the breadth of our review.

## Results

### Selection of sources of evidence

**Figure 1.**
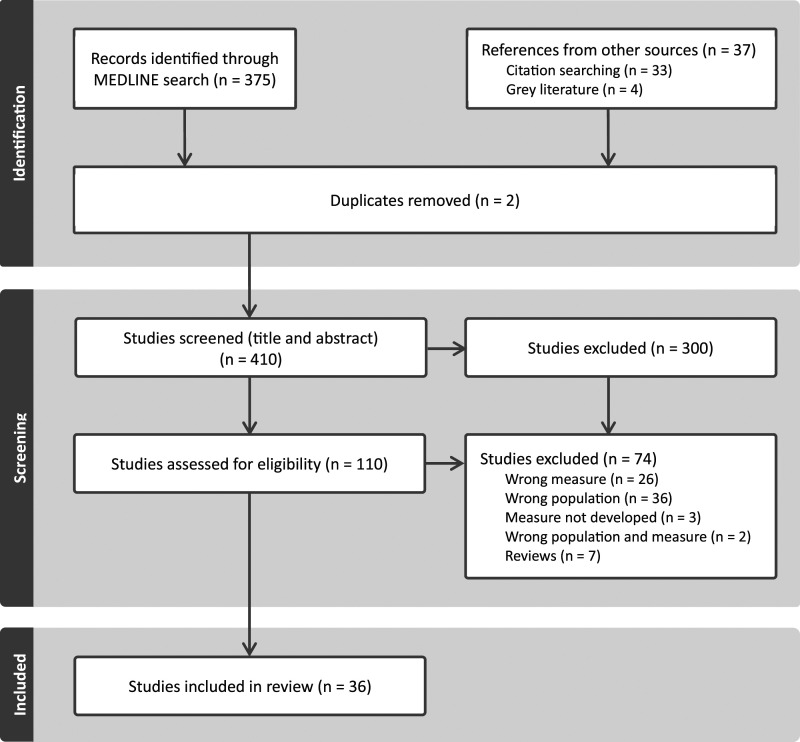
PRISMA flow diagram.

An initial total of 412 studies were identified; 375 studies from the Medline search, 4 articles found through grey literature, and 33 studies identified through citation searching. Following the removal of 2 duplicates, 410 articles were title and abstract screened. One hundred and ten studies were assessed as eligible for full-text review and of these, 74 were excluded for reasons including incorrect population, incorrect measure (measure of patient satisfaction rather than experience), review of measures (reference lists were screened), or the measure not yet being developed (protocols or qualitative analyses of focus groups as part of methodology in developing a PREM). A final 36 studies were included in the scoping review. Figure 1 shows an overview of this selection process.

### Characteristics of sources of evidence

The studies included in this review are dated from 2000 to 2023. The studies were conducted in 19 countries and in 17 languages, with the majority being conducted in England (8 studies) and in English (18 studies). Study designs were quantitative (*n* = 29) or mixed methods (*n* = 7) and were most commonly scale development (*n* = 18) or cross-sectional studies (*n* = 11). The most common purpose of the studies was the development and validation of a patient-reported experience measure (*n* = 26), assessment of differences in reported experiences between groups (*n* = 5), and assessment of the impact of different healthcare interventions and services on care experience (*n* = 3). Of the studies included, nine assessed paediatric patient experiences, 26 assessed mental health care experiences, one included a measure for people with cognitive impairment, and one included a PREM specifically designed for people with dementia. The number of participants included in the studies ranged from 11 to 17,762.

The studies included 32 different PREMs (including different versions of a generic-PREM adapted for a range of contexts: e.g., inpatient, outpatient, and forensic settings) that had been used with vulnerable populations. The PREMs ranged in terms of number of items included (5–69 items), number of scale dimensions (1–9 dimensions), and response options (2-to-7-point Likert scales alongside free-text responses). PREMs were completed in various formats including in-person (paper, digital, and interview), via email, mail, and telephone. Recall periods ranged from during hospitalisation to 24 months following healthcare experience. Table 1 contains characteristic details of individual studies included.

**Table 1.** Overview of the included studies.

Author (Year), country	PREM	Language	Study design	Outcome	Category	Target population	Participants	Dimensions	Items	Response options	Administration	Recall period
Bal et al. (2020), Canada	PREM for children in Urgent and Emergency care	English	Cross-sectional	Determined differences in reported experience between children and parents	Paediatric	Children presenting to ED (median age = 11yrs)	109	6	21	4 options: yes definitely, yes to some extent, no, don’t know/can’t remember	In-person, via email, phone	In ED or day after
Berzina et al. (2021), Latvia	Psychiatric inpatient patient experience questionnaire on-Site (PIPEQ-OS)	Latvian	Cross-sectional	Outlined areas for improvement in service delivery and correlates to satisfaction	Mental health	People who are inpatients on psychiatric wards	419	3	21	5pt Likert scale from ‘not at all’ to ‘to a very large extent’	In-person paper	One day before discharge
Bjertnaes et al. (2015), Norway	Psychiatric inpatient patient experience questionnaire on-Site (PIPEQ-OS)	Norwegian	Scale development	Developed and validated PIPEQ-OS.	Mental health	People who are inpatients on psychiatric wards	552	3	21	5pt Likert scale from ‘not at all’ to ‘to a very large extent’	In-person paper	Patients on site at week 37 of 2014
Bjørngaard et al. (2007), Norway	Psychiatric Out-patient experiences questionnaire (POPEQ)	Norwegian	Cross-sectional	Determined impact of different outpatient teams and clinics on patient experience scores	Mental health	Mental health outpatient service users	6570	3	11	5pt Likert scale from ‘not at all’ to ‘to a very large extent’	Mailed	Within a month
Bruyneel et al. (2018), Belgium	Flemish patient survey of mental healthcare	Dutch	Scale development	Developed and validated PREM for people accessing mental health services	Mental health	Mental health service users	5168	9	35	4pt Likert scale from ‘never’ to ‘always’	-	At least 4 days inpatient or at least 4 contacts outpatient or community
Chen et al. (2023), Taiwan	Chen et al.'s patient-centred care questionnaire	Taiwanese	Cross-sectional	Critical factors of patient centred care predicted satisfaction	Mental health	People with schizophrenia	150	5	-	-	In-person (self-completed or read by interviewer)	During hospitalisation
Currie et al. (2020), Canada	Patient experience survey for Addiction and mental health	English	Scale development	Developed and validated patient experience measure for addiction and mental health programs	Mental health	Mental health and addiction service users	1222	6	30	4 options: yes definitely, yes to some extent, no, don’t know/can’t remember	-	-
Danielson et al. (2008), US	Quality of care measure, patient form (QOC-P)	English	Scale development	Developed and validated PREM for psychiatric inpatients to assess efficacy of implementing Engagement model in units	Mental health	People who are inpatients on psychiatric units	81	3	23	5pt Likert scale from ‘strongly disagree’ to ‘strongly agree’	Paper	During stay
Delaney et al. (2015), US	Combined assessment of psychiatric environments (CAPE)	English	Scale development	Developed and validated PREM for evaluation of person-centred care on inpatient psychiatric units	Mental health	People who are inpatients on psychiatric units	150	5	24	4pt Likert scale from ‘never’ to ‘always’	In-person paper (assistants could help read)	At least 4 days inpatient, within 24 hours to discharge
Forouzan et al. (2014), Iran	Mental health System responsiveness questionnaire (MHSRQ)	Farsi	Scale translation	Translated PREM into Farsi and tested with patients of mental health services in Iran	Mental health	Outpatient mental health service users	500	8	40	4pt Likert scale from ‘never’ to ‘always’	In-person paper (assistants could help read)	Attended appointment in last 12 months
Garratt et al. (2006), Norway	Psychiatric Out-patient experiences questionnaire (POPEQ)	Norwegian	Scale development	Developed and validated PREM for people accessing outpatient mental health services	Mental health	Outpatient mental health service users	6677	3	11	5pt Likert scale from ‘not at all’ to ‘to a very large extent’	Mailed	Reviewed series of apps not just most recent
Gensichen et al. (2011), Germany	Patient assessment of chronic illness care questionnaire (PACIC)	German	Cross-sectional	Assessed the psychometric characteristics of the PACIC in primary care patients with major depression	Mental health	Primary care service users with major depression	442	5	20	5pt Likert scale from ‘almost never’ to ‘almost always’	Mailed	Services received in last 6 months
Gigantesco et al. (2003), Italy	Rome Opinion questionnaire for psychiatric Wards (ROQ-PW)	Italian	Scale development	Developed and validated measure for assessing care in inpatient psychiatric wards	Mental health	People who are inpatients on psychiatric ward	169	3	12	5pt Likert scale (wording changes for items)	In-person paper	6–7 days after admission
Hester et al. (2015), Ireland	Service user quality of CareE (SEQUenCE)	English	Scale development	Developed and validated measure for assessing care in mental health services	Mental health	People with bipolar affective disorder or psychotic disorder attending mental health services	60	-	40	5pt Likert scale from ‘strongly disagree’ to ‘strongly agree’	-	Services received in last 12 months
Hopwood and Tallett (2011), England	Hopwood and Tallett’s paediatric PREM	English	Scale development	Developed and validated paediatric PREM for outpatient clinics	Paediatric	Paediatric outpatients (age > 8yrs)	3442	-	34	-	Mailed	-
Iyer et al. (2023), Canada and India	Show Me you care	English, French, Tamil	Scale development	Developed a tool to understand patient and family experience of services for psychosis	Mental health	People with psychosis or schizophrenia	230	-	9	7pt Likert scale from ‘strongly disagree’ to ‘strongly agree’	-	2 years of therapy (completed at months 3, 12, and 24)
Jerrell (2006), US	Mental health Statistics improvement program (MHSIP) Adult consumer survey	English	Cross-sectional	Assessed psychometric characteristics of questionnaire with people with severe mental illness accessing public mental health system	Mental health	People with severe mental illness who have accessed public mental health system	459	3	21	-	In-person interview	-
Jones et al. (2022), US	Jones et al.'s paediatric patient experience questionnaire	English	Cross-sectional	Provided insight into children’s experiences of neuropsychological assessment	Paediatric	Children undergoing neuropsychology assessments	167	-	15	5pt Likert scale	iPad	Immediately after assessment
Karisalmi et al. (2020), Finland	Karisalmi et al.'s paediatric patient experience questionnaire	Finnish	Case–control study	Determined influence of medical clowning on children’s hospital experience	Paediatric	Children >4yrs in hospital for pre-operative canula insertion	70	3	5	Varied	Digitally on tablet	Following operation
Lundqvist et al. (2014), Denmark	Quality in psychiatric care- forensic inpatient (QPC-FIP)	Danish	Scale translation	Translated scale and validated with Danish forensic inpatient population	Mental health	People in forensic psychiatric care	139	7	34	4pt Likert scale from ‘totally disagree’ to ‘totally agree’	-	In ongoing forensic psychiatric care
Mavaddat et al. (2009), England	Patient experience questionnaire (PEQ)	English	Scale development	Developed and validated PREM for assessing primary mental healthcare	Mental health	People with mental health concerns who have attended primary care clinic	217	-	20	5pt Likert scale from ‘strongly disagree’ to ‘strongly agree’	Mailed	Attended primary care in last 3 months
McGrath et al., 2024, Ireland	Experience of service questionnaire (ESQ)	English	Cross-sectional	Determined differences in reported experience between children and parents	Paediatric	Children with ADHD	44	-	15	3pt Likert scale and 3 free text	-	Following clinical review
Olsen et al. (2010), Norway	Psychiatric Out-patient experiences questionnaire (POPEQ)	Norwegian	Rasch analysis	Conducted a Rasch analysis on the POPEQ to support the validity of the scale	Mental health	People who attended outpatient psychiatric service	17762	3	11	5pt Likert scale from ‘not at all’ to ‘to a very large extent’	Mailed	0–2 months
Reynolds et al. (2021), Austria, Estonia, Germany, Netherlands, Norway, Spain, UK	P3CEQ	Catalan, Dutch, English, Estonian, German, Norwegian, Spanish	Cross-sectional	Assessed usability and quality of PREM for use across multiple countries and services for older people	Cognitive impairment, mental health	Older people with complex care needs who live at home and were integrated service users	26, 71	2	11	tick boxes and open responses	In-person	-
Ruud et al. (2023), Norway	Patient experiences in psychiatric departments for the elderly (PEPDE)	Norwegian	Scale development	Developed and validated PREM for older people in psychiatric departments	Mental health	Older people in psychiatric departments	151	4	20	5pt Likert scale from ‘not at all’ to ‘to a very large extent’	In-person (read aloud and scored by interviewer)	Ready for discharge after stay of min 7 days
Ryberg et al. (2023), Denmark	“What do you think of hospital? Help us to get better?”	Danish	Scale translation	Translated PREM into Danish and pre-tested with 23 children and adolescents	Paediatric	Children attending outpatient services (8–17yrs)	11	-	-	3pt Likert scale from ‘never’ to ‘always’	In-person (paper or interview)	Immediately following appointment
Schröder et al. (2007), Sweden	Quality in psychiatric care - 2 (QPC-2)	Swedish	Scale development	Developed and validated measure for assessing care for people accessing inpatient psychiatric care	Mental health	People accessing inpatient psychiatric services	116	5	69	4pt Likert scale from ‘totally disagree’ to ‘totally agree’	In-person paper	On discharge
(Schröder et al., 2010), Sweden	Quality in psychiatric care - inpatient (QPC-IP)	Swedish	Scale validation	Assessed the psychometric properties of the QPC-IP with psychiatric inpatient population	Mental health	People accessing inpatient psychiatric services	265	6	30	4pt Likert scale from ‘totally disagree’ to ‘totally agree’	In-person paper	On discharge
Schröder et al. (2011), Sweden	Quality in psychiatric care - outpatient (QPC-OP)	Swedish	Scale development	Developed and validated measure for assessing care for people accessing outpatient psychiatric care	Mental health	People attending outpatient psychiatric clinics	1340	8	32	4pt Likert scale from ‘totally disagree’ to ‘totally agree’	In-person paper	Immediately after appointment
Serhal et al. (2020), Canada	Serhal et al.’s Telepsychiatry experience questionnaire	English	Scale development	Developed and validated scale to measure experience of telepsychiatry	Mental health	People accessing telepsychiatry	274	4	18	5pt Likert scale from ‘strongly disagree’ to ‘strongly agree’	-	Immediately following appointment
Sidey-Gibbons et al. (2019), UK	Sidey-Gibons et al.’s patient reported experience measure	English	Scale development	Developed and validated measure for physical health planning for mental health users and their carers	Mental health	Mental health service users and their carers	267	6	19	5pt Likert scale from ‘strongly disagree’ to ‘strongly agree’	Online or paper	-
Sugarman (2016), England	Sugarman’s PREM for people with dementia	English	Scale development	Developed and validated PREM for people living with moderate to advanced dementia	Dementia	Older people with moderate to advanced dementia accessing community services	16	-	5	yes or no with images	In-person interview (point at answers on paper)	During care experience
Teksoz et al. (2017), Turkey	Patient’s nursing care Perception tool	Turkish	Pre/post mixed methods	Creative play intervention influenced children’s experience of care from nurses	Paediatric	Children attending inpatient services (8–12yrs)	30	-	15	5pt Likert scale from ‘strongly disagree’ to ‘strongly agree’	-	-
Webb et al. (2000), England	Your treatment and care assessment tool	English	Cross-sectional	Assessed care experiences across psychiatric services	Mental health	People accessing mental health services	503	5	24	-	In-person	-
Williams et al. (2018), UK	Williams et al.’s paediatric PREM	English	Cross-sectional	Assessed experiences of children and carers with epilepsy services	Paediatric	Paediatric epilepsy service users (age = 5–14yrs mostly)	692	-	14	5pt Likert scale from ‘strongly disagree’ to ‘strongly agree’	-	Over 12 month period
Wray et al. (2018), England	“What do you think of hospital? Help us to get better!”	English	Scale development	Developed and piloted PREMs for young people in hospitals and outlined barriers to routine use of PREMs for YP.	Paediatric	Children attending inpatient and outpatient services (age = 8–11 yrs)	143	3	-	3pt Likert scale from ‘never’ to ‘always’	In-person paper	At discharge or immediately after appointment

*Note.* Response Option describes most common response scale used in the PREM.

### Synthesis of results

Across the 32 PREMs, 81 different dimensions were included in the measures. The most common construct across the PREMs was the effectiveness of care, for example “has your doctor or psychologist been able to help you?” (Ruud et al., 2023), which was included in 10 PREMs. Dimensions relating to the care environment and patient involvement and participation in their own care were also recorded in nine PREMs. Other frequently included constructs were provision of information, relationships with staff, and safety. Table 2 contains details of the most common constructs included across the PREMs.

**Table 2.** Dimensions reported in PREMs for vulnerable populations.

PREMs included	Dimensions														
	Access and timeliness	Autonomy	Care coordination	Communication	Confidentiality, privacy, and rights	Dignity and respect	Discharge and follow-up	Effectiveness of care	Emotional support	Environment	Information	Patient involvement and participation	Person-centred care	Relationships with staff	Safety
Chen et al.’s patient-centred care questionnaire	-	✔	✔	✔	-	-	-	-	✔	-	✔	-	-	-	-
Combined assessment of psychiatric environments (CAPE)	-	✔	-	-	-	✔	-	✔	-	-	-	✔	-	-	✔
Experience of service questionnaire (ESQ)	-	-	-	-	-	-	-	-	-	-	-	-	-	-	-
Flemish patient survey of mental healthcare	-	-	✔	-	✔	-	✔	✔	-	-	✔	-	✔	✔	✔
Hopwood and Tallett’s paediatric PREM	-	-	-	-	-	-	-	-	-	-	-	-	-	-	-
Jones et al. ’s paediatric patient experience questionnaire	-	-	-	-	-	-	-	-	-	-	-	-	-	-	-
Karisalmi et al.’s paediatric patient experience questionnaire	-	-	-	-	-	-	-	-	✔	-	-	-	-	-	-
Mental health Statistics improvement program (MHSIP) Adult consumer survey	✔	-	-	-	-	-	-	✔	-	-	-	-	-	-	-
Mental health System responsiveness questionnaire (MHSRQ)	✔	✔	-	✔	✔	✔	-	✔	-	✔	-	-	-	-	-
P3CEQ	-	-	✔	-	-	-	-	-	-	-	-	-	✔	-	-
Patient assessment of chronic illness care questionnaire (PACIC)	-	-	-	-	-	-	✔	-	-	-	-	✔	-	-	-
Patient experience questionnaire (PEQ)	-	-	-	-	-	-	-	-	-	-	-	-	-	-	-
Patient experience survey for Addiction and mental health	✔	-	✔	✔	-	-	-	✔	-	-	-	-	✔	✔	-
Patient experiences in psychiatric departments for the elderly (PEPDE)	-	-	-	-	✔	-	-	✔	✔	-	✔	-	✔	-	✔
Patient’s nursing care Perception tool	-	-	-	-	-	-	-	-	-	-	-	-	-	-	-
PREM for children in Urgent and Emergency care	✔	-	-	✔	✔	✔	-	-	✔	✔	✔	✔	-	-	-
Psychiatric inpatient patient experience questionnaire on-Site (PIPEQ-OS)	-	-	-	-	-	-	-	✔	-	-	-	✔	-	-	-
Psychiatric Out-patient experiences questionnaire (POPEQ)	-	-	-	-	-	-	-	✔	-	-	✔	-	-	✔	-
Quality in psychiatric care - 2 (QPC-2)	-	-	-	-	-	✔	-	✔	-	✔	-	✔	-	-	✔
Quality in psychiatric care - inpatient (QPC-IP)	-	-	-	-	-	-	✔	-	✔	✔	-	✔	-	-	✔
Quality in psychiatric care - outpatient (QPC-OP)	✔	✔	-	-	-	-	✔	-	✔	✔	✔	✔	-	-	-
Quality in psychiatric care- forensic inpatient (QPC-FIP)	-	-	-	-	-	-	✔	-	✔	✔	-	✔	--	-	✔
Quality of care measure, patient form (QOC-P)	-	-	-	-	-	-	-	-	-	✔	-	✔	-	✔	-
Rome Opinion questionnaire for psychiatric Wards (ROQ-PW)	-	-	-	-	-	-	-	-	-	✔	✔	-	-	✔	-
Serhal et al.’s Telepsychiatry experience questionnaire	✔	-	-	-	-	-	-	✔	-	-	-	-	-	-	✔
Service user quality of CareE (SEQUenCE)	-	-	-	-	-	-	-	-	-	-	-	-	-	-	-
Show Me you care	-	-	-	-	-	-	-	-	-	-	-	-	-	-	-
Sidey-Gibons et al.’s patient reported experience measure	-	-	✔	-	-	-	-	-	-	-	-	-	✔	✔	-
Sugarman’s PREM for people with dementia	-	-	-	-	-	-	-	-	-	-	-	-	-	-	-
“What do you think of hospital? Help us to get better!”	-	-	-	-	-	-	-	-	-	✔	-	-	-	✔	-
Williams et al.’s paediatric PREM	-	-	-	-	-	-	-	-	-	-	-	-	-	-	-
Your treatment and care assessment tool	-	-	-	-	-	-	-	-	-	-	✔	-	-	✔	-

One of the aims of the scoping review was to evaluate the selected PREMs in respect to the adaptions made for vulnerable populations. Twenty-one of the PREMs (65.6%) made adaptions to the measure being used to improve usability of the instruments for vulnerable populations. The most frequent adaptions were simplification of wording and sentence structure, with such adaptions being reported for 16 of the PREMs. Other common adaptions included shortening the length of the PREM, the use of visual aids (e.g., facial emojis and other icons included in questionnaires to support independent responding), and assistance reading or completing the PREM. The adaptions made to the PREMs are outlined in Table 3.

**Table 3.** Adaptions to the PREMs for vulnerable populations.

PREMs included	Adaptions								
	PREM adapted (yes or no)	Wording/sentence structure simplified	Engaging colours and images used	Visual aids included	Item scales/response options simplified	Scale shortened	Assistance to read/complete	Layout and question order improved	Other adaptions
Chen et al.’s patient-centred care questionnaire	Yes	-	-	-	-	-	✔	-	-
Combined assessment of psychiatric environments (CAPE)	Yes	✔	-	-	-	-	-	-	-
Experience of service questionnaire (ESQ)	No	-	-	-	-	-	-	-	-
Flemish patient survey of mental healthcare	Yes	✔	-	-	-	-	-	-	-
Hopwood and Tallett’s paediatric PREM	Yes	✔	✔	-	✔	✔	-	✔	-
Jones et al.'s paediatric patient experience questionnaire	Yes	✔	-	✔	-	-	-	-	Touch-screen, read aloud function
Karisalmi et al.’s paediatric patient experience questionnaire	Yes	-	-	✔	-	-	✔	-	-
Mental health Statistics improvement program (MHSIP) Adult consumer survey	Yes	-	-	-	-	-	✔	-	-
Mental health System responsiveness questionnaire (MHSRQ)	Yes	✔	-	-	-	-	-	-	-
P3CEQ	Yes	-	-	-	-	-	✔	-	-
Patient assessment of chronic illness care questionnaire (PACIC)	No	-	-	-	-	-	-	-	-
Patient experience questionnaire (PEQ)	Yes	✔	-	-	-	✔	-	-	-
Patient experience survey for Addiction and mental health	Yes	✔	-	-	-	-	-	-	-
Patient experiences in psychiatric departments for the elderly (PEPDE)	Yes	✔	-	✔	-	✔	-	✔	-
Patient’s nursing care Perception tool	No	-	-	-	-	-	-	-	-
PREM for children in Urgent and Emergency care	No	-	-	-	-	-	-	-	-
Psychiatric inpatient patient experience questionnaire on-Site (PIPEQ-OS)	Yes	-	-	-	-	-	✔	-	-
Psychiatric Out-patient experiences questionnaire (POPEQ)	No	-	-	-	-	-	-	-	-
Quality in psychiatric care - 2 (QPC-2)	Yes	✔	-	-	-	-	-	-	-
Quality in psychiatric care - inpatient (QPC-IP)	No	-	-	-	-	-	-	-	-
Quality in psychiatric care - outpatient (QPC-OP)	Yes	✔	-	-	-	-	-	-	-
Quality in psychiatric care- forensic inpatient (QPC-FIP)	Yes	✔	-	-	-	-	-	-	-
Quality of care measure, patient form (QOC-P)	No	-	-	-	-	-	-	-	-
Rome Opinion questionnaire for psychiatric Wards (ROQ-PW)	Yes	✔	-	-	-	-	-	-	-
Serhal et al.’s Telepsychiatry experience questionnaire	No	-	-	-	-	-	-	-	-
Service user quality of CareE (SEQUenCE)	Yes	✔	-	-	-	-	-	✔	-
Show Me you care	Yes	✔	-	-	-	✔	-	-	-
Sidey-Gibons et al.’s patient reported experience measure	No	-	-	-	-	-	-	-	-
Sugarman’s PREM for people with dementia	Yes	✔	-	✔	✔	✔	✔	-	-
“What do you think of hospital? Help us to get better?”	Yes	✔	✔	✔	✔	-	-	✔	Paper copy
Williams et al.’s paediatric PREM	No	-	-	-	-	-	-	-	-
Your treatment and care assessment tool	No	-	-	-	-	-	-	-	-

A secondary aim of the scoping review was to evaluate which PREMs have been psychometrically validated for use with vulnerable populations. Construct validity was the most frequently assessed type of validity (22 PREMs), followed by face validity (17 PREMs), and content validity (15 PREMs). Three PREMs assessed criterion validity and one assessed the measure’s responsiveness. In respect to reliability, internal consistency was reported for 23 PREMs and test-retest for 10 PREMs. The psychometric properties that were assessed for specific PREMs can be found in Table S2 (available as supplemental material).

## Discussion

### Summary of evidence

As a move towards person-centred care is seen in healthcare, it is imperative to understand and include the experiences of people with dementia. There are currently no appropriate PREMs designed specifically for people with moderate to advanced dementia available for use across healthcare contexts. Therefore, as a preliminary step in developing a PREM that is suitable for this population, we have identified 32 PREMs that have been used across 36 studies with vulnerable populations, including people with mental health concerns, cognitive impairment, and children.

Within the identified PREMs, the most frequently identified dimensions were the effectiveness of care, the care environment, and patient involvement and participation in their own care. The effectiveness of care being the most frequently identified dimension reflects the overlap between patient-reported experience measures, patient-reported outcome measures, and measures of patient satisfaction. While our criteria aimed to include measures that captured patient experiences, we did not exclude measures based upon their inclusion of other constructs such as outcomes of care or satisfaction with care. This lack of clarity surrounding the constructs being measured has been identified within the literature (Bull, 2021). While the effectiveness of care and satisfaction with care are often related to care experiences (Black et al., 2014; Lapin et al., 2019; Mendlovic et al., 2022), the conflation of these constructs reduces the ability to use a specific PREM to determine tangible changes that can be made to improve care experiences. As part of the next steps in our development process, we will engage with stakeholders to determine whether the dimensions outlined in the PREMs used with other vulnerable populations reflect the priorities of people with dementia in improving their experiences of care.

As part of this scoping review, we outlined the adaptions made to PREMs to ensure their appropriateness for vulnerable populations. The most common adaptions made to the PREMs involved the simplification of wording or sentence structure. However, 11 PREMs (34.4%) had no reported adaptions made to ensure their appropriateness and usability for their target populations (seven PREMs were used in mental health contexts and four PREMs were used in paediatric settings). This review also found a lack of use of technology as part of the adaptions to the PREMs. The PREM developed by Jones et al. (2022) that was used with children who had undergone neuropsychological assessments was the only instrument that specifically mentioned the use of technology in ensuring the appropriateness of the PREM for their target population. The PREM involved a touch screen and read-aloud function that children could activate if required to promote independent responding. The use of features such as these, alongside other considerations of how technology can be used to support populations with varying cognitive and communicative abilities, should be considered in future development of PREMs for vulnerable populations.

Within our review we did not specify levels of severity for PREMs used in mental health services and thus, contexts varied from inpatient psychiatric services for complex presentations (Bjertnaes et al., 2015; Ruud et al., 2023) to attendance of an outpatient appointment at primary health clinics (Gensichen et al., 2011; Olsen et al., 2010). The Patient Assessment of Chronic Illness Care (PACIC) (Gensichen et al., 2011), which reported no adaptions, was used to assess experiences of care in primary health clinics for people with depression. It is likely that no adaptions to the PACIC were required in this setting. In contrast, settings treating people with other mental health conditions may have found that the PACIC was not appropriate. While our review aimed to cast a wide net in terms of considering a range of mental health contexts, PREMs that possessed less adaptions or were used with populations possessing less cognitive and communicative barriers may be of lesser utility in developing a PREM for people with moderate to advanced dementia.

A recent scoping review of the adaptions made to quality of life measures for people with sensory impairments, communication challenges or changes to cognitive capacity found similar adaptions (e.g., simplification of wording, item reduction, use of images) had been made (Milte et al., 2023, 2023b). It is possible that similar adaptations may be effective in increasing completion of PREMs by people with moderate to advanced dementia and may be a valuable starting point for future work in this space.

Our review found one PREM that had been specifically developed for use with people with moderate to advanced dementia (Sugarman, 2016). Sugarman’s (2016) thesis included piloting a PREM with 16 people with moderate to advanced dementia who accessed a community service that included a memory clinic and post-diagnostic supports. The PREM was used by occupational therapists, community psychiatric nurses, psychiatry registrars, and a consultant psychiatrist. Following the pilot, Sugarman (2016) interviewed staff who had trialled the PREM to determine the measure’s effectiveness. It was noted that the use of the PREM by the staff member who had provided care could increase socially desirable responding by patients due to a power imbalance. Staff suggested that carers may be well-positioned to administer the PREM however, this could also influence responses. Feedback also described a need for more flexible and tailored administration of the PREM as some staff found the PREM to work better in a more conversational format, some patients felt patronised by overly simplified language, and patients varied in terms of whether the use of visual aids assisted in responding or added an extra level of complexity to the task. Overall, while the PREM was used successfully in some circumstances, there is a need for further consideration of the challenges of administering a PREM for people with moderate to advanced dementia prior to wider-scale implementation.

In recent years, the Consumer Choice Index – Six Dimension (CCI-6D) has been developed and validated for measurement of the quality of care in long-term care (Milte et al., 2019). Given the high percentage (54%) of people with dementia residing in long-term care facilities (Australian Institute of Health and Welfare, 2023), the use of the CCI-6D may also be an appropriate instrument for understanding the care experiences of people with moderate to advanced dementia. In the initial development of the CCI-6D, participants with advanced cognitive impairments had carers participate as proxy (Milte et al., 2019) however, Milte et al. (2023, 2023b) have recently demonstrated the validity of the instrument for people with dementia in residential aged care. Given the specificity of the CCI-6D to the aged care context (dimensions include care time, shared spaces, own room, outside and gardens, meaningful activities, and care flexibility), this measure could be adapted to also account for care experiences in other healthcare services.

### Limitations

As aforementioned, our review was limited by the overlaps between the different constructs of patient experience, patient outcomes, and patient satisfaction. While we endeavoured to include measures that possessed items related to experience, not all included PREMs were purely measures of patient experience, and they often included the related yet distinct constructs of care outcomes and satisfaction.

A large number of PREMs that may have included dimensions relevant to vulnerable populations were also excluded during our screening process as they were often paediatric PREMs that were completed by parental proxy reports rather than being adapted to younger children’s ability level. This reliance on proxy reports in paediatric research reflects a similar approach to understanding the experiences of people with dementia. However, given the evidence that carers do not always possess the same priorities or opinions on care experiences as the patients they are representing (Bal et al., 2020; Griffiths et al., 2020; Sands et al., 2004), it is necessary to strive towards more inclusive research and consider innovative ways of adapting measures to become more accessible for vulnerable populations.

Another limitation of our scoping review is that we only conducted our search strategy in Medline. However, our intention was to scope the literature and we did supplement this with a search of the grey literature alongside citation searching to ensure a thorough search of the literature. We included a date restriction on our search which may have also limited our study selection. This choice was made based upon the premise that the development and use of PREMs in healthcare is relatively new and thus, we predominantly wanted to focus our search on contemporary literature.

Finally, we excluded studies not written in English and the majority of our included studies were conducted in Western countries. While we will pilot our PREM in an Australian healthcare context, for future generalisability it is worth considering that across different cultures, different elements of care may be prioritised.

## Conclusions

Our scoping review has outlined multiple PREMs that have been used with vulnerable populations. PREMs varied greatly in terms of number of participants, number of items and scale response options, dimensions included, response format, and recall period. The majority of identified PREMs (26 PREMs) examined experiences of care in mental health settings where cognition and communication may be less impacted. The most frequent dimension included in the identified PREMs was the effectiveness of care, highlighting a gap in the literature regarding measures that focus purely on care experiences (rather than outcomes or satisfaction) for vulnerable populations. Other frequent constructs included in PREMs were care environment and patient involvement and participation in their own care. Existing instruments have limited adaptions for vulnerable populations, predominantly outlining adaptions involving the simplification of wording and sentence structure. While measures of care experiences for people with dementia have been trialled (Milte et al., 2023, 2023b; Sugarman, 2016), development of a PREM specifically for people with moderate to advanced dementia that includes relevant dimensions of care, appropriate and flexible adaptions, and applicability across healthcare contexts is required. Such a measure will be key to understanding and monitoring self-reported healthcare experiences for people with moderate to advanced dementia, ensuring that consumer voices are elevated and platformed to inform quality improvement.

## Supplemental Material

Supplemental Material - Patient-reported experience measures for people living with dementia: A scoping review Supplemental Material for Patient-reported experience measures for people living with dementia: A scoping review by Madison Chapman, Rachel Milte, Suzanne Dawson and Kate Laver in Dementia.
