# Supplementary material for: Patient-reported experience measures for people living with dementia: A scoping review
Source: Dementia (London). 2024 Aug 8;23(8):1354–81. doi: 10.1177/14713012241272823 (PMC11475968; doi:10.1177/14713012241272823)
Supplement: Supplemental Material - Patient-reported experience measures for people living with dementia: A scoping review [file sj-pdf-1-dem-10.1177_14713012241272823.pdf]

SUPPLEMENTARY MATERIAL

Tables

Table S1

PREMs Database Search on MEDLINE

Search Number	Text	Results
1	‘patient reported experience measure’.mp.	131
2	‘patient experience’.mp. or ‘satisfaction with care’.ti,ab.	12945
3	child*.mp. or adolescent*.ti,ab.	2905977
4	cognit*.mp. and impair*.ti,ab.	160783
5	(dementia or alzheimer*).mp. or delirium.ti,ab.	320672
6	Down Syndrome.ti,ab.	16946
7	'mental health'.mp.	272556
8	(psychosis or psychotic or schizophreni*).mp.	225402
9	measure.mp. or tool.ti,ab.	1300364
10	2 and 9	2066
11	1 or 10	2152
12	3 or 4 or 5 or 6 or 7 or 8	3689310
13	11 and 12	375

Note: Ovid MEDLINE(R) and Epub Ahead of Print, In-Process, In-Data-Review & Other Non-Indexed Citations, Daily and Versions <1946 to September 01, 2023>

SUPPLEMENTARY MATERIAL

Table S2

Psychometric properties of PREMs for vulnerable populations

PREMs Included	Reliability		Validity				Responsiveness
	Internal consistency	Test-retest	Face validity	Content validity	Construct validity	Criterion validity	
Chen et al's Patient-Centred Care Questionnaire	α = 0.70-0.86	-	-	✓	-	-	-
Combined Assessment of Psychiatric Environments (CAPE)	α = 0.91	r = 0.76	✓	✓	✓	-	-
Experience of Service Questionnaire (ESQ)	-	-	-	-	-	✓	-
Flemish Patient Survey of Mental Healthcare	-	-	✓	✓	✓	✓	-
Hopwood and Tallett's Paediatric PREM	-	-	✓	-	-	-	-
Jones et al's Paediatric Patient Experience Questionnaire	-	-	-	-	-	-	-
Karisalmi et al's Paediatric Patient Experience Questionnaire	-	-	-	-	-	-	-
Mental Health Statistics Improvement Program (MHSIP) Adult Consumer Survey	α = 0.73 - 0.81	r = 0.45 - 0.61	-	-	✓	-	-
Mental Health System Responsiveness Questionnaire (MHSRQ)	α = 0.56 - 0.92	-	✓	✓	✓	-	-
P3CEQ	α = 0.55-0.76	-	-	-	✓	-	-
Patient Assessment of Chronic Illness Care questionnaire (PACIC)	α = 0.91	-	-	-	✓	-	-
Patient Experience Questionnaire (PEQ)	α = 0.94	r = 0.86	✓	-	✓	-	-
Patient Experience Survey for Addiction and Mental Health	α = 0.77-0.85	r = 0.53-0.82	✓	-	✓	✓	-
Patient Experiences in Psychiatric Departments for the Elderly (PEPDE)	α > 0.64	-	✓	✓	-	-	-
Patient's Nursing Care Perception Tool	α = 0.92	-	-	✓	✓	-	✓
PREM for Children in Urgent and Emergency Care	-	-	✓	-	-	-	-
Psychiatric Inpatient Patient Experience Questionnaire On-Site (PIPEQ-OS)	α = 0.79-0.91	ICC = 0.83-0.84	✓	-	✓	-	-
Psychiatric Out-Patient Experiences Questionnaire (POPEQ)	α > 0.8	ICC > 0.75	✓	✓	✓	-	-
Quality in Psychiatric Care - 2 (QPC-2)	α = 0.98	-	✓	✓	-	-	-
Quality in Psychiatric Care - Inpatient (QPC-IP)	α = 0.96	-	-	-	✓	-	-
Quality in Psychiatric Care - Outpatient (QPC-OP)	α = 0.95	-	-	-	✓	-	-
Quality in Psychiatric Care - Forensic Inpatient (QPC-FIP)	α = 0.96	-	✓	✓	✓	-	-
Quality of Care Measure, Patient Form (QOC-P)	α = 0.89	-	-	✓	✓	-	-

SUPPLEMENTARY MATERIAL

Rome Opinion Questionnaire for Psychiatric Wards (ROQ-PW)	α = 0.82	k > 0.6	✓	✓	✓	-	-
Serhal et al's Telepsychiatry Experience Questionnaire	RRC > 0.72	-	-	✓	✓	-	-
Service user Quality of CareE (SEQUenCE)	α = 0.87	r = 0.65	✓	✓	✓	-	-
Show Me You Care	α = 0.91	ICC = 0.78	✓	-	✓	-	-
Sidey-Gibons et al's Patient Reported Experience Measure	marginal r = 0.87	r = 0.7	-	-	✓	-	-
Sugarman's PREM for people living with dementia	-	-	✓	✓	✓	-	-
"What do you think of hospital? Help us to get better!"	-	-	✓	✓	-	-	-
Williams et al's Paediatric PREM	-	-	-	-	-	-	-
Your Treatment and Care Assessment Tool	α = 0.89	-	-	-	✓	-	-
